# Effects of Fine Particulate Matter (PM_2.5_) on Systemic Oxidative Stress and Cardiac Function in ApoE^−/−^ Mice

**DOI:** 10.3390/ijerph13050484

**Published:** 2016-05-12

**Authors:** Yiling Pei, Rongfang Jiang, Yunzeng Zou, Yu Wang, Suhui Zhang, Guanghe Wang, Jinzhuo Zhao, Weimin Song

**Affiliations:** 1 Department of Environmental Health, School of Public Health and Key Laboratory of Public Health Safety, Fudan University, Shanghai 200032, China; 12111020012@fudan.edu.cn (Y.P.); rfjiang@fudan.edu.cn (R.J.); 2 Department of Cardiology, Shanghai Institute of Cardiovascular Diseases, Zhongshan Hospital, Fudan University, Shanghai 200032, China; zou.yunzeng@zs-hospital.sh.cn; 3 Pharmacology and Toxicology Department, Shanghai Institute for Food and Drug Control, Shanghai 201203, China; ohyue@163.com (Y.W.); 12111020016@fudan.edu.cn (S.Z.); 4 Department of Hygienic Toxicology, College of Public Health, Harbin Medical University, Harbin 150081, China; 10111020016@fudan.edu.cn

**Keywords:** PM_2.5_, atherosclerosis, autonomic nervous system (ANS), echocardiography (Echo), oxidative stress

## Abstract

Aim: In this study, we aimed to explore the toxic mechanisms of cardiovascular injuries induced by ambient fine particulate matter (PM_2.5_) in atherosclerotic-susceptible ApoE^−/−^ mice. An acute toxicological animal experiment was designed with PM_2.5_ exposure once a day, every other day, for three days. Methods: ApoE^−/−^ and C57BL/6 mice were randomly categorized into four groups, respectively (*n* = 6): one control group, three groups exposed to PM_2.5_ alone at low-, mid-, and high-dose (3, 10, or 30 mg/kg b.w.). Heart rate (HR) and electrocardiogram (ECG) were monitored before instillation of PM_2.5_ and 24 h after the last instillation, respectively. Cardiac function was monitored by echocardiography (Echo) after the last instillation. Biomarkers of systemic oxidative injuries (MDA, SOD), heart oxidative stress (MDA, SOD), and NAD(P)H oxidase subunits (p22phox, p47phox) mRNA and protein expression were analyzed in mice. The results showed that PM_2.5_ exposure could trigger the significant increase of MDA, and induce the decrease of heart rate variability (HRV), a marker of cardiac autonomic nervous system (ANS) function with a dose–response manner. Meanwhile, abnormal ECG types were monitored in mice after exposure to PM_2.5_. The expression of cytokines related with oxidative injuries, and mRNA and protein expression of NADPH, increased significantly in ApoE^−/−^ mice in the high-dose group when compared with the dose-matched C57BL6 mice, but no significant difference was observed at Echo. In conclusion, PM_2.5_ exposure could cause oxidative and ANS injuries, and ApoE^−/−^ mice displayed more severe oxidative effects induced by PM_2.5_.

## 1. Introduction

In the 1930s, the first epidemiological study on the relationship between high levels of air pollutants and cardiovascular disease (CVD) [1] was published. Then broad investigations revealed that exposure to PM_2.5_ was linked to the development of atherosclerosis in humans [2,3,4,5], but potential mechanisms still remain unclear regarding the effects in susceptible populations. Atherosclerosis is a chronic process, and PM_2.5_ exposure involved in disease progression is not only associated with the production of long-term atherogenesis but also associated with cardiovascular effects (myocardial infarction or cardiovascular death) through acute exposure. Although several studies have explained the partial contribution of PM_2.5_ chronic exposure to the current atherosclerotic state, studies with short exposure are far from enough [6].

The previous assessment of ambient particulate matter pollution did not include a risk above a concentration of 100 µg/m^3^ for PM_2.5_, because in developed countries the level of PM_2.5_ is very low. However, the level of PM_2.5_ in China during the winter was much higher, and the daily average concentration of PM_2.5_ even exceeds 200 µg/m^3^, and it usually lasts for 3–7 days. Furthermore, the chemical composition and concentration of air PM_2.5_ differs significantly between China and most developed countries. As a result, in our study, animals were exposed to high levels of PM_2.5_ without invasive procedures (intratracheal instillation) once a day, every other day, for three days.

PM_2.5_ exposure is a risk factor for CVD via mechanisms that likely include pulmonary and systemic inflammation, oxidative injuries, immune injuries, accelerated atherosclerosis, and altered cardiac autonomic function. The main mechanism by which PM_2.5_ contributes to increased cardiac risk is by initiating and promoting atherosclerotic progression, the underlying cause of most cardiovascular diseases [7]. In a series of animal experiments, apolipoprotein E knockout mice (ApoE^−/−^ mice) was used as the animal model for establishing the atherosclerosis model. In our previous study, results already showed that ApoE^−/−^ mice exhibited systematic inflammation and immune injuries after exposure to PM_2.5_ [8]. In the present study, ApoE^−/−^ mice were exposed to PM_2.5_ by instillation. This exposure paradigm allowed us to explore the oxidative injuries and cardiac function induced by acute exposure to PM_2.5_.

### New and Noteworthy

Acute exposure to PM_2.5_ was associated with cardiovascular injuries in atherosclerosis model ApoE^−/−^ mice. Dysfunction of ANS, Echo, and oxidant stress might be partial of the biological mechanisms linking PM_2.5_ and atherosclerosis. This study is the very first time cardiac function of PM_2.5_ exposure on cardiovascular system in a susceptible mouse model by Echo in China has been investigated.

## 2. Materials and Methods

### 2.1. Animals

Twenty-four eight-week-old male ApoE^−/−^ mice (21–25 g) and twenty-four age-matched male C57BL/6 mice were purchased from the Beijing Laboratory Animal Center, Chinese Academy of Science. Immediately after arrival, the ApoE^−/−^ and C57BL/6 mice were weighed and randomly allocated into four subgroups (*n* = 6), respectively. Animals were kept under specific pathogen-free (SPF)-conditions for a total duration of two months. Animals were housed at constant temperature (21 °C ± 1 °C) and relative humidity (60%) under a regular light/dark schedule (light 7:00 a.m. to 7:00 p.m.). Food and water were freely available. The ApoE^−/−^ mice received a Western diet (21% fat, 0.15% cholesterol, Institute of Laboratory Animal Science, Chinese Academy of Medical Sciences) for eight weeks, starting from eight weeks of age, to establish the atherosclerotic model. C57BL/6 mice received a normal diet for eight weeks. All the animal studies were approved by the Ethics Committee of Animal Care and Experimentation of the National Institute for Environmental Studies, China (2014-01-GWXY-SWM-01).

### 2.2. PM_2.5_ Sampling

The specific method was taken according to the report by Wang *et al.* [9]. PM_2.5_ was collected with a Thermo Anderson G-2.5 air sampler (Model GV 2630 Series, Austin, TX, USA) onto the glass fiber filters from 1 June to 31 October 2013 in Shanghai, China. Before sampling, the filters were heated at 200 °C for 24 h and, after sampling, filters were cut into small pieces and immerged in 0.9% saline, followed by sonicating 3 × 10 min with a sonicator (Model JL-120DT, Nanjing, China). The PM_2.5_ suspension was treated by vacuum-freeze drying, and then the concentrated components were weighd and stored at −20 °C. Fine particles should be diluted with sterilized 0.9% physiological saline according to experimental concentration and stored at 4 °C within 24 h before instillation.

### 2.3. Elemental and Ionic Analysis in Fine Particles

Fine particles were collected onto PTFE (Teflon) filters for metallic element and ion analysis. The elemental analysis for fine particles was carried out by an ICP-OES/7300DV instrument (Optim, Darmstadt, Germany) and 17 elements were detected. Dionex3000 ion chromatograph (Dionex, Bannockburn, IL, USA) was used to analyze the ion components.

### 2.4. PM_2.5_ Exposure

At the age of 16 weeks, ApoE^−/−^ and C57BL/6 mice were randomized into four subgroups (*n* = 6), respectively. The mice were instilled with 0.9% saline or fine particles of 15 μL/10 g body weight (b.w.) respectively, and the exposure groups were categorized as control, low-, mid- and high-dose(0, 3, 10 and 30 mg/kg b.w.), respectively. The instillation was conducted once a day, and every other day, in total for three days.

### 2.5. Electrocardiograph (ECG) Monitoring and Heart Rate Variability (HRV) Assessment

Before instillation of PM_2.5_ and after 24 h of the last instillation, animals were briefly anesthetized by 10% chloral hydrate (intraperitoneal injection, i.p.), with spontaneous breathing and steady heart beats maintained, electrocardiograph (ECG) morphology of mice were recorded for 20 min, respectively. A model SMUP-E4 Bioelectric Signals Processing system (Jia long educational instrument factory, Shanghai, China) automatically recorded the ECG dates, and only stable segments were used for HRV analysis. In our study, 256-beat segment of HRV indices were calculated, the frequency range for power in the low-frequency (LF) power and high-frequency (HF) power for mice is defined as 0.04–0.15 Hz and 0.15–0.4 Hz, respectively, and the ratio between LF and HF (LF/HF) [9].

### 2.6. Echocardiography and Hemodynamic Measurements

Transthoracic echocardiography was performed after 24 h post-exposure of the third instillation using a 30-MHz high-frequency scan head (Visual Sonics Vevo770, Visual Sonics, Toronto, ON, Canada). Internal temperature was maintained at 37 °C, as mice were continuously sedated with 1% isoflurane (in 100% O_2_) during assessment. Echocardiograph detection included the evaluation of heart rate, left ventricular end diastolic dimension (LVED*_d_*), left ventricular end-systolic dimension (LVES*_d_*), left ventricular ejection fraction (LVEF), fractional shortening (FS), left ventricular end diastolic anterior wall thickness (LVAW*_d_*), and left ventricular end-diastolic posterior wall thickness (LVPW*_d_*).

### 2.7. Biochemical Analysis of Serum

Twenty-four hours after the last tracheal instillation, animals were briefly anesthetized by chloral hydrate (10%, i.p.) via abdomen for recording the ECG and Echo results. Then, the blood was collected in a tube to clot for 2 h at room temperature and centrifuged at 3000 rpm for 10 min at 4 °C. The serum was collected and stored at −80 °C for the biochemical analysis. The cardiac tissues were collected for determining the mRNA and protein expression. The contents of malondialdehyde (MDA) and super oxide dismutase (SOD) activity in serum were assayed by a commercial colorimetric assay kit (Nanjing Jiancheng Bioengineering Institute, Jiangsu, China).

### 2.8. Reverse Transcriptase Polymerase Chain Reaction (RT-PCR)

The heart was immediately excised and divided into two parts through vertical section. One part of the heart was snap frozen in liquid nitrogen and stored at −80 °C until analysis, and the other part was conserved in 4% formaldehyde for histopathological evaluation. Frozen ventricle tissue samples were homogenized in TRIzol reagent (Invitrogen, Waltham, MA, USA), and total RNA was extracted from the tissue according to the manufacturer’s suggested protocol. The total mRNA was converted into cDNA by reverse transcriptase polymerase chain reaction (rt-PCR) by using the PrimeScript^TM^ RT reagent Kit (TaKaRa, Kusatsu, Japan) according to the supplied protocol. The cDNA generated was used as a template in subsequent real-time PCR analyses. cDNA was amplified by SYBR green master mix (TaKaRa, Kusatsu, Japan) using a real-time quantitative polymerase chain reaction system (ABI7500 thermocycler, Foster City, CA, USA). We measured two components of the NADPH oxidase, including mice p22phox and p47phox. The gene-specific mice primers were designed from previous studies [9,10] (Table 1). Cycling conditions were as following: step 1, 30 s at 95 °C; step 2, 5 s at 95 °C; step 3, 30–34 s at 60 °C; with step 2 to step 3 repeated 40 times. The 2−∆CT method was used to normalize transcription to GAPDH mRNA and calculate the fold induction relative to control. The relative mRNA levels in the exposure groups were standardized in order to obtain fold increases in mRNA related to the level in the control group.

### 2.9. Western Blot

The total proteins of the left ventricle were lysed in RIPA lysis buffer, which contained a protease inhibitor PMSF (Amresco Scientific, Solon, OH, USA). Protein concentration was quantified using a protein assay kit (BCA, Shanghai, China). Samples containing 50 μg of proteins were put separately on a 12% SDS-PAGE gel and transferred to nitrocellulose filter membranes (Millipore Darmstadt, Germany). NC membranes were blocked for 2 h at room temperature with 2%–5% BSA, reacted with p22 antibody and p47 antibody (1:500, Santa Cruz Biotechnology, Dallas, TX, USA), and anti-GAPDH antibody (1:1000, Kangchen Biotech, Shanghai, China) at 4 °C overnight, and then incubated with a secondary HRP-conjugated antibody (1:2000, Fermentas Biotech, Burlington, ON, Canada) or 1 h at room temperature. After incubation with the secondary antibody, the blots were detected with ECL (Millipore, Darmstadt, Germany). The bands obtained were then densitometrically analyzed using “Quantity One” software (Bio-Rad, Hercules, CA, USA). The ratios were compared using the expression of the bands in a ratio against GAPDH expression for the same sample.

## 3. Statistical Analysis

All of the data were expressed as the means ± standard deviation (x ± SD). The differences between control and PM_2.5_ treated groups were analyzed using statistical software (SPSS 16.0, Chicago, IL, USA) with a two-way ANOVA (PM_2.5_ doses or mice groups as factors) and the Tukey’s multiple comparison. For the purpose of the study, we have adhered to three kinds of comparisons, which are clearly defined in the figure captions under the figures. The level of *p* < 0.05 was defined as statistically significant.

## 4. Results

### 4.1. Elemental and Ionic Contents of Fine Particles

The results of elemental and ionic concentration of PM_2.5_ were shown in Table 2. The results showed that the concentration of certain metal elements (Ca, Fe, K, Na, and Al) and ions (SO_4_^2−^, NH_4_^+^, and NO_3_^−^) was much higher than other elements.

### 4.2. Heart Rate (HR) and HRV Indices Analyses

The C57BL/6 and ApoE^−/−^mice were randomly divided into four groups, respectively. Before instillation of PM_2.5_ and after 24 h of the third instillation, ECG morphology of mice was recorded for 20 min respectively. After three applications of instillation, the HR decreased significantly in the high-dose (30 mg/kg b.w.) group in C57BL/6 and ApoE^−/−^ mice compared with the control group. A similar pattern of HR was also found in the C57BL/6 mice after exposure to PM_2.5_. For HRV indices (Table 3), after three applications of instillation, LF showed a significant decrease in high-dose PM_2.5_ exposure group in ApoE^−/−^ mice, and medium-dose, high-dose PM_2.5_ exposure in C57BL/6 mice. High-dose PM_2.5_ exposure in ApoE^−/−^ mice and C57BL/6 mice causes a significant increase in HF when compared with control. For the ratio between LF and HF (LF/HF), there was a significant decrease in groups of C57BL/6 and ApoE^−/−^ at a high dose in a dose–response manner. After three times of instillation, we found that the changes for LF, HF and LF/HF in ApoE^−/−^ and C57BL/6 mice were almost the same with the exposure with the decrease of LF and LF/HF and increase of HF becoming more significant. As for the changes in total variation (TV), significant decreases were observed in C57BL/6 and ApoE^−/−^ mice exposed to high-dose PM_2.5_ when compared to the control group. Simultaneously, two-way ANOVA verified that groups and PM_2.5_ had no significant interaction, but there were main effects of mice and PM_2.5_ on the activity of LF and LF/HF (^c^ *p* < 0.05). 

### 4.3. Electrocardiogram (ECG) Type Changes

**Figure 1:**
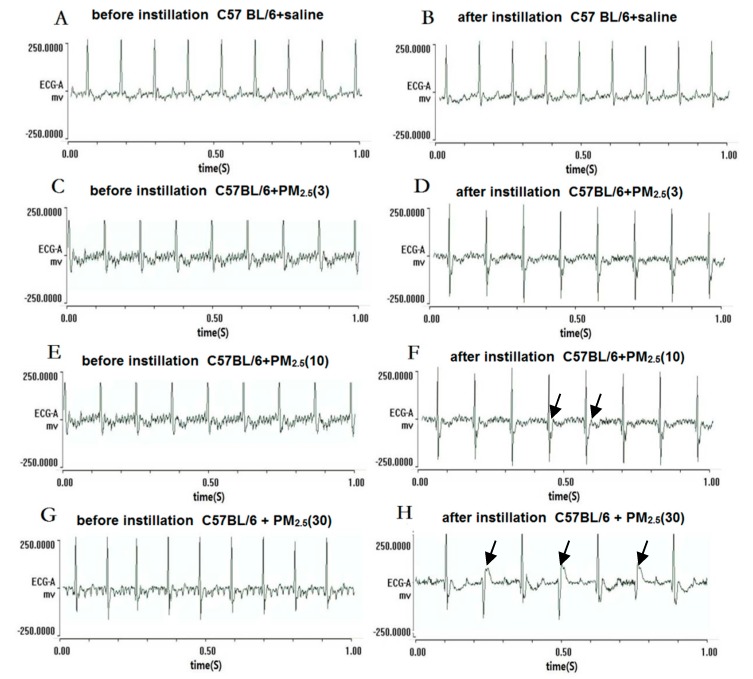
Electrocardiogram (ECG) types before instillation and after instillation in C57BL/6 mice. (**A**,**C**,**E**,**G**) represent the ECG in the mice before PM_2.5_ treatment; (**B**,**D**,**F**,**H**) represent the ECG in the mice after PM_2.5_ treatment, the doses were 0, 3, 10, and 30 mg/kg b.w., respectively; (**F**) black arrows represent ST-segment depression; and (**H**) black arrows represent ventricular escape rhythm.

**Figure 2:**
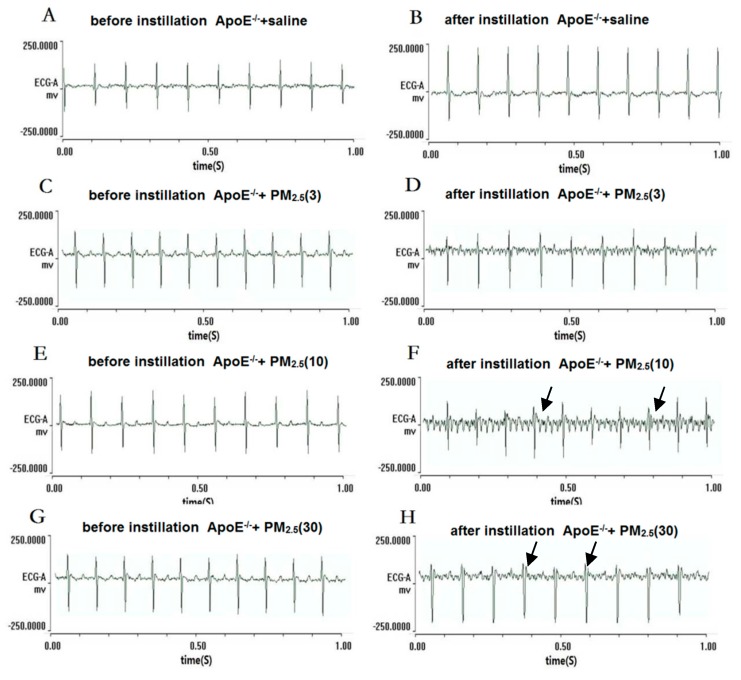
Electrocardiogram (ECG) types before instillation and after instillation in ApoE^−/−^ mice. (**A**,**C**,**E**,**G**) represent the ECG in the mice before PM_2.5_ treatment; (**B**,**D**,**F**,**H**) represent the ECG in the mice after PM_2.5_ treatment, the doses were 0, 3, 10, and 30 mg/kg b.w., respectively; (**F**) black arrows represent left bundle-branch block; and (**H**) black arrows represent right bundle-branch block (RBBB).

Normal sinus rhythm and HR were observed in the mice of the control group at the end of saline treatments (Figure 1B and Figure 2B). In low-dose PM_2.5_ exposure C57BL/6 and ApoE^−/−^ mice, there were no significant changes of the ECG type and the average HR was slightly lower than the control group (Figure 1D and Figure 2D). In other experimental groups, abnormal ECG was observed in different PM_2.5_ treatment groups, such as ST-segment depression (Figure 1F) and ventricular escape rhythm (Figure 1H). Right bundle-branch block (RBBB) was observed in ApoE^−/−^ mice exposed to high-dose PM_2.5_ (Figure 2H). Additionally, the C57BL/6 and ApoE^−/−^ mice exposed to high-dose PM_2.5_ showed the partial conduction block which were the main characteristics of abnormal ECG.

### 4.4. Echocardiography Changes

**Figure 3:**
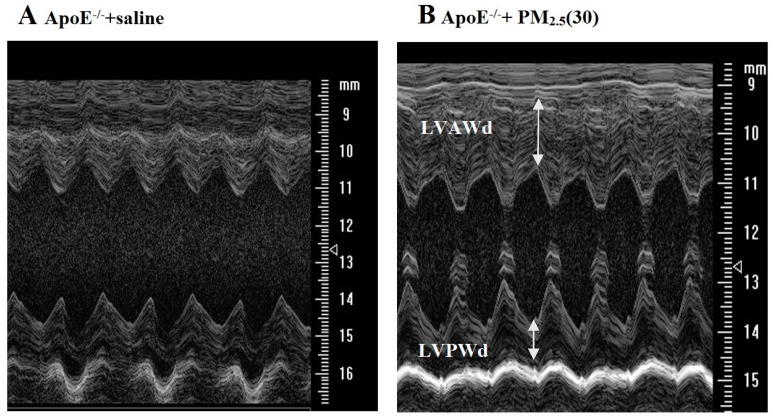
Echocardiography images of ApoE^−/−^ mice. (**A**) Represent control group, normal structure; (**B**) represent ApoE^−/−^ mice was exposed to high doses PM_2.5_ (30 mg/kg b.w.). White arrows represent LVAW*_d_* and LVPW*_d_* increased significantly in ApoE^−/−^ mice exposed to high-dose PM_2.5_, respectively.

Echocardiography was performed after PM_2.5_ exposure (Table 4). The C57BL/6 and ApoE^−/−^ mice were randomly divided to four groups (*n* = 6), respectively. Two-way ANOVA verified that groups and PM_2.5_ had no significant interaction, and the results showed that LVEF and FS increased significantly in ApoE^−/−^ mice exposed to low-dose and medium-dose PM_2.5_ compared to the dose-matched C57BL/6 mice; whereas LVAW*_d_* and LVPW*_d_* increased significantly just in the high-dose PM_2.5_ exposure group. As a matter of fact, only two of six ApoE^−/−^ mice exposed to high-dose PM_2.5_ showed the increased of LVAW*_d_* and LVPW*_d_*, indicating myocardial hypertrophy (Figure 3), whereas the rest of them were similar with the control group. Quantitative analysis revealed that there were no significant difference of LVED*_d_* and LVES*_d_* between high -dose PM_2.5_ exposure ApoE^−/−^ mice and the dose-matched C57BL/6 mice.

### 4.5. Measurement of Oxidants and Antioxidants in the Plasma

**Figure 4:**
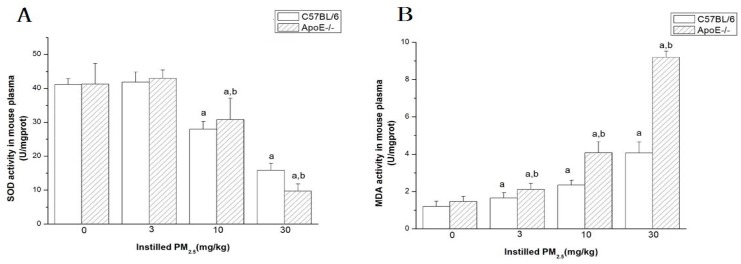
The levels of SOD and MDA in the plasma of mice (*n* = 6) after exposed to PM_2.5_. (**A**) represents SOD activity in plasma; (**B**) represents MDA activity in plasma. The doses were 0 (control), 3, 10, and 30 mg/kg b.w., respectively. Significant difference (^a^ *p* < 0.05) between PM_2.5_ group and control group, and significant difference (^b^ *p* < 0.05) between C57BL/6 and dose-matched ApoE^−/−^ mice were observed.

The levels of SOD and MDA in the plasma were measured (Figure 4). SOD are antioxidant enzymes, which can protect the individual against the oxidant stress. SOD activity decreased significantly in C57BL/6 and ApoE^−/−^ mice at medium-dose and high-dose PM_2.5_ in comparison with the control group. SOD activity was inhibited by 63% and 78% in the ApoE^−/−^ and C57BL6 mice exposed to the high-dose PM_2.5_. Moreover, in high-dose PM_2.5_ exposed mice, SOD activity in ApoE^−/−^ mice decreased significantly than dose-matched C57BL6 groups. On the other hand, the contents of MDA had a significant increase in the mice exposed to PM_2.5_ with a dose-trend relationship. Additionally, ApoE^−/−^ mice exposed to PM_2.5_ also had a significant increase in MDA when compared to C57BL6 at the low-, medium-, and high-dose PM_2.5_.

### 4.6. mRNA Expressions of p22phox and p47phox in Myocardium

**Figure 5:**
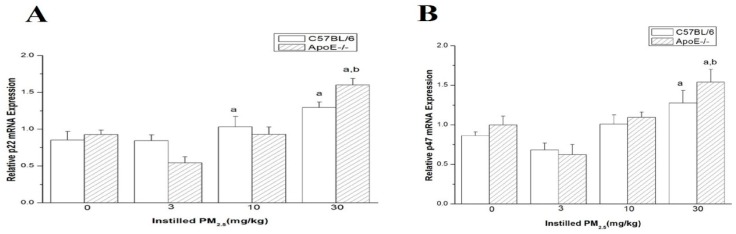
The mRNA expression of p22phox and p47phox in myocardium of mice after exposed to PM_2.5_. (**A**) represents the relative p22phox mRNA expression in mice; (**B**) represents the relative p47phox mRNA expression in mice. The doses were 0.0 (control), 3, 10, and 30 mg/kg b.w. Significant difference (^a^ *p* < 0.05) between PM_2.5_ group and the control group, and significant difference (^b^ *p* < 0.05) between C57BL/6 and dose- matched ApoE^−/−^ mice were observed.

The mRNA expression for two of the NAD(P)H oxidase subunits (p22phox, p47phox) increased significantly in C57BL/6 and ApoE^−/−^ mice exposed to high-dose PM_2.5_ in comparison with the control group (Figure 5). Moreover, both p22phox and p47phox mRNA expressions increased significantly in high-dose exposure ApoE^−/−^ mice compared with myocardium dose-matched C57B/L6 mice (*n* = 6).

### 4.7. Protein Expression of p22phox and p47phox in Myocardium

**Figure 6:**
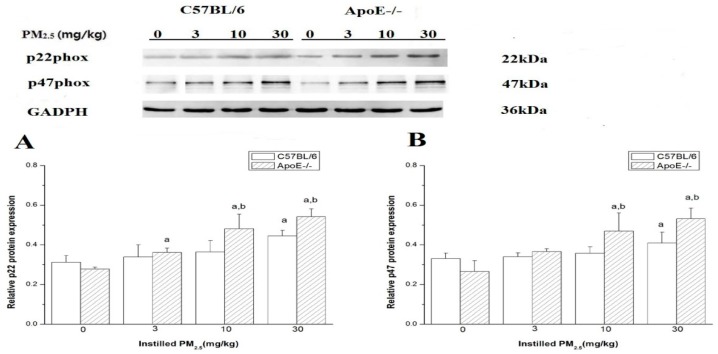
Protein expression of p22phox and p47phox in mice myocardium exposed to PM_2.5_ by Western blot. (**A**) represents the relative p22phox protein expression in mice; (**B**) represents the relative p47phox protein expression in mice. The doses were 0 (control), 3, 10, and 30 mg/kg b.w. Significant difference (^a^ *p* < 0.05) between PM_2.5_ group and control group, and significant difference (^b^ *p* < 0.05 ) between C57BL6 and dose- matched ApoE^−/−^ mice were observed.

The results showed that the protein expression of p22phox and p47phox elevated in left ventricle of C57BL/6 and ApoE^−/−^ mice after exposure to PM_2.5_ in a dose-dependent manner (Figure 6). The protein levels of p22phox and p47phox in the high-dose group were higher than that in the control group (*p* < 0.05). Both p22phox and p47phox protein expression increased significantly in the myocardium of ApoE^−/−^ mice exposed to medium-dose and high-dose PM_2.5_ compared with that from myocardium dose-matched C57BL/6 mice (*n* = 6).

### 4.8. Pathological Characters of Heart Tissue and Vessel Tissue

**Figure 7:**
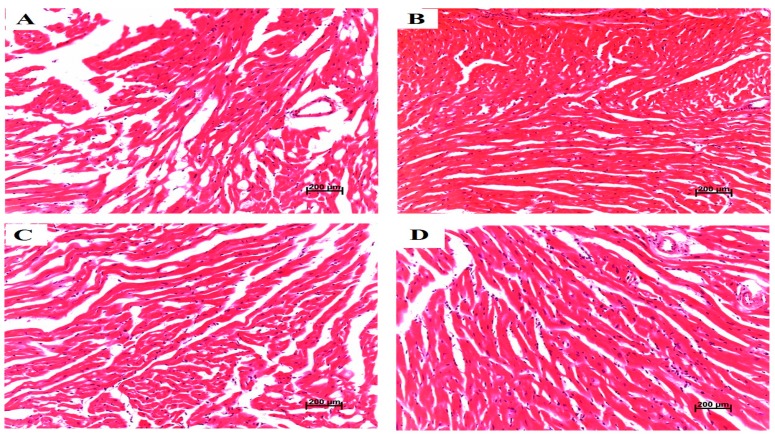

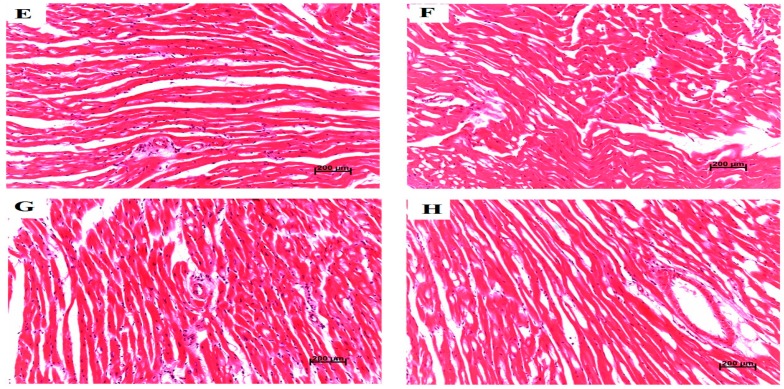
Left ventricle histological evaluation and pathological scores in mice after exposed to PM_2.5_. (**A**–**D**) represent histological lesions (×200) of left ventricle in C57BL/6 mice exposed to PM_2.5_ in dose of 0 (control), 3, 10, and 30 mg/kg b.w., respectively; (**E**–**H**) represent histological lesions (×200) of left ventricle in ApoE^−/−^ mice exposed to PM_2.5_ in dose of 0 (control), 3, 10, and 30 mg/kg b.w., respectively.

**Figure 8:**
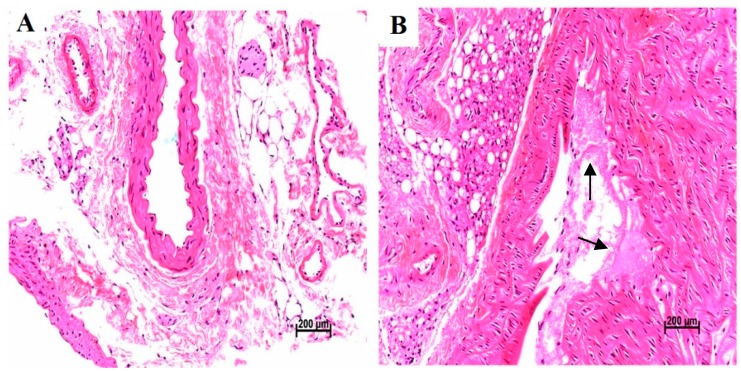
Coronary artery histological evaluation and pathological scores in mice after exposed to fine particles. (**A**) represents normal vessel tissue in C57BL/6 mice; (**B**) black arrows represent plague formation in ApoE^−/−^ mice. Histological lesions (×200) of coronary arteries in mice.

Histological evaluations of left ventricle indicated that there were no detectable cardiac histological changes in enroll mice (Figure 7). Sixteen-week-old ApoE^−/−^ mice fed a high-cholesterol diet had plaque formation in the coronary artery, which establishes the atherosclerosis model (Figure 8).

## 5. Discussion

To identify that the toxic mechanisms of PM_2.5_ induced cardiovascular injuries in human with atherosclerosis, we established an ApoE^−/−^ mice model. ApoE^−/−^ mice start to develop atherosclerosis lesions from stage II while having been fed the Western diet for 8–10 weeks, with the mice being started on the diet when they are between 4–8 weeks old [11]. In our study, ApoE^−/−^ mice were fed with a Western diet for eight weeks when the mice were eight weeks old. Histological evaluations of mice coronary arteries indicated that the atherosclerosis progress in the ApoE^−/−^ mice was between stage I and II. We conducted PM_2.5_ exposure specifically on 16-week ApoE^−/−^ mice in order to examine the toxicological effect of instillation exposure to PM_2.5_ on the development of atherosclerosis at initial stages. The morphological feature of early stage (stage I and II) lesions in ApoE^−/−^ mice are very similar to those found in people [11].

Considering the association between PM_2.5_ toxicity and cardiovascular injuries, we utilized three doses of PM_2.5_. The specific calculating method was referred to the study of Thomson *et al.* [12]. In our study, the exposure concentration of PM_2.5_ used was 10 times higher than the american air quality standards set by the EPA in 1997 (24 h averaging time, PM_2.5_, 65 μg/m^3^), and the anticipated cumulative dose based on 9% deposition and a minute-volume of 36 mL for a 30 g mouse would be 3 mg/kg b.w. after three times instillation. The doses of PM_2.5_ was concordant with our previous study, which showed that ApoE^−/−^ mice exhibited cardiovascular and pulmonary injuries after exposure to 3, 10, and 30.0 mg/kg of PM_2.5_ in a dose-dependent manner [8].

After three times instillation_,_ the results from ECG showed that HR, LF/HF, LF, and TV decreased, and HF increased in response to medium and high-dose PM_2.5_ in the C57BL/6 group. In the dose–trend relationship between HRV and PM_2.5_ concentration, ApoE^−/−^ plus high-dose PM_2.5_ caused a significant increase in HF when compared with control. Several studies have demonstrated that the influence of PM could involve an imbalance in cardiac autonomic control. Among the frequency-domain parameters of HRV, HF reflects the cardiac parasympathetic activity, and LF is reported to reflect both the cardiac sympathetic and parasympathetic nervous activities [13,14]. Additionally, LF/HF reflects the balance of cardiac autonomic nervous (sympathetic and parasympathetic) control [15]. The present findings were consistent with those investigations in humans which decrease in HR and the relationship between LF and HF (LF/HF) were found in response to inhaled PM_2.5_ [16,17,18]. A decrease in HRV was associated with poor prognosis in patients with cardiovascular disease and may trigger adverse cardiovascular effect (myocardial infarction or cardiovascular death) [19,20,21,22,23]. On the other hand, we also found that exposure to PM_2.5_ induced abnormal ECG types, such as ST segment depression in C57BL/6 mice and right bundle branch block in ApoE^−/−^ mice. However, normal sinus rhythm and HR were observed in most mice of the control group at the end of saline treatments by self-control. The abnormal ECG types ST segment depression was related to ischemia and hypoxia in the myocardium induced by PM_2.5_, and a conduction block was associated with severe cardiovascular disease [24]. In addition, ventricular escape rhythm may occur as a result of ischemic heart disease, or metabolic imbalances, due to decrease in cardiac output, will lead to atrial kick, palpitations, weakness, or dizziness [25]. Similar results were also found in epidemiological studies that PM predicted risk elevated for ST-segment depression in subjects with coronary artery disease [26,27]. Briefly, imbalance of cardiac autonomic nervous control and myocardium ischemia and hypoxia effects may provide insights into an underlying mechanism of cardiovascular disease resulting from exposure to PM_2.5_.

Transthoracic echocardiographic analyses revealed systolic function EF and FS were significantly increased in ApoE^−/−^ mice plus low-dose and medium-dose PM_2.5_ exposure compared to dose-matched C57BL/6 mice, but no difference in the high-dose group. LVAW*_d_* and LVPW*_d_* increased significantly in ApoE^−/−^ mice plus high-dose PM_2.5_ indicted that ApoE^−/−^ mice have cardiac hypertrophy induced by increased collagen deposition. In another study where mice were exposed to high particulate concentrations of diesel exhaust (300 ug/m^3^) for five days a week also found increased collagen deposition, but no significant reduction in cardiovascular function in adult mice [28]. Sun *et al.* [29] found that long term exposure to PM_2.5_ during early life can cause significant cardiovascular dysfunction at adulthood. Two recent studies only focus on long-term exposure. However, in our present study, short exposure to PM_2.5_ can cause slight cardiovascular dysfunction in ApoE^−/−^ mice plus low-dose and medium-dose, the mechanism may be that there is microcirculatory compensation to atherosclerotic disease of increasing severity, so at the low and medium-dose PM_2.5_ exposure, cardiac systolic functions in ApoE^−/−^ mice were increased compared with the control group.

Our results demonstrated clearly the increase of MDA and the decrease of antioxidase SOD in PM_2.5_ exposure, which were in a dose–response manner to show the rising imbalance of antioxidant and oxidant abilities in the circulatory system. Similar results were demonstrated in other studies that pulmonary and heart tissue oxidative stress were also observed in mice, and most research approves the oxidative stress initiated by reactive oxygen species (ROS) [9]. NAD(P)H oxidase is the major source of ROS, and our research showed that NAD(P)H oxidase subunits p22phox and p47phox expression in hearts increased on the mRNA and protein levels. Comparisons of p22phox and p47phox between treatment with C57BL/6 plus PM_2.5_ and the same dose of the ApoE^−/−^ group were statistically different, according to which we believe that the effect of PM_2.5_ might influence ApoE^−/−^ more. Elemental analysis in PM_2.5_ clearly showed that the concentration of certain metal elements, such as Cu, Fe, Zn, and Pb, were higher than other elements, which has proven to be associated with the generation of ROS and oxidative injury [30,31]. ROS can be generated from the surface of particles where transition metals (Cu, Fe, Zn, Pb) are absorbed that catalyzing Fenton’s reaction (Fe^2+^ + H_2_O_2_ + H^+^ → Fe^3+^ + OH· + H_2_O) generate the highly-reactive hydroxyl radical able to induce oxidative damage [32,33]. Then, in the presence of high ROS formation, mitochondrial damage with the induction of NADPH-oxidase units does occur together with an activation of inflammatory cells and increased numbers of macrophages capable of ROS and reactive nitrogen species generation [34,35]. The underlying mechanisms responsible for the association between fine PM exposure and impaired HRV are not yet fully understood. Oxidative stress may mediate PM-related effects on cardiovascular function and ECG [36]. Wan found that long inhalation exposure to PM results in upregulation of the potential inflammatory mediator visfatin, which led to oxidant stress in ApoE^−/−^ mice after PM_2.5_ challenge [37].

## 6. Conclusions

In this study, acute exposure to PM_2.5_ was associated with increased oxidative stress, an imbalance in cardiac autonomic control, and an abnormal ECG type of myocardial ischemia and hypoxia in mice. At the high PM_2.5_ exposure, ApoE^−/−^ mice are more susceptible to oxidative effects, but no significant differences were observed in cardiac dysfunction between C57BL/6 and ApoE^−/−^ mice. Hence, the toxicological effects of PM_2.5_ on human health were mediated by oxidant stress mechanisms, which might be dependent on PM_2.5_ concentration and its constituents.

## Figures and Tables

**Table 1:** Sequence of primers.

Genes	Primer Pairs
p22phox-m-F	CATCGTGGCTACTGCTGGAC
p22phox-m-R	TGGACCCCTTTTTCCTCTTT
p47phox-m-F	CGAAGAAGCCTGAGACATACC
p47phox-m-R	ATATCCCCTTTCCTCACCACC
GADPH-m-F	ACCACAGTCCATGCCATCAC
GADPH-m-R	TCCACCACCCTGTTGCTGTA

**Table 2:** Elemental and ionic concentration of PM_2.5_.

Elements	Concentration (%)	Elements	Concentration (%)	Elements	Concentration (%)
Ca	21.6	Ba	0.4	SO_4_^2−^	40.6
Fe	21.5	Cu	0.4	NH_4_^+^	27.1
K	21.1	As	0.3	NO_3_^−^	18.9
Na	14.9	Sr	0.1	Cl^−^	9.2
Al	6.3	Ti	0.1	Na^+^	2.2
Zn	5.3	V	0.1	K^+^	1.8
Mg	4.3	Cr	0.1	C_2_O_4_^2−^	0.8
Pb	2.1	Ni	0.05	F^−^	0.1
Mn	1.2				

**Table 3:** Changes in heart rate and HRV indices in experimental groups.

Groups	HR (bmp)	TV	LF (ms^2^)	HF (ms^2^)	LF/HF
Before instillation					
C57BL/6 + saline	516 ± 25	5.82 ± 3.66	2.45 ± 1.11	1.87 ± 0.97	1.4 ± 0.37
C57 BL/6 + PM_2.5_ (3)	533 ± 31	4.73 ± 2.48	2.29 ± 0.32	2.08 ± 0.45	1.16 ± 0.40
C57BL/6 + PM_2.5_ (10)	505 ± 25	5.77 ± 2.76	2.7 ± 1.40	2.07 ± 1.00	1.52 ± 0.82
C57 BL/6 + PM_2.5_ (30)	504 ± 19	5.05 ± 1.97	2.92 ± 0.94	2.34 ± 1.06	1.32 ± 0.32
After instillation					
C57 BL/6 + saline	508 ± 23	5.61 ± 2.05	2.61 ± 0.90	1.87 ± 0.75	1.44 ± 0.36
C57BL/6 + PM_2.5_ (3)	494 ± 34 ^b^	3.93 ± 1.78 ^b^	2.02 ± 0.42	2.15 ± 0.35	0.93 ± 0.07 ^a^
C57BL/6 + PM_2.5_ (10)	475 ± 49 ^b^	3.24 ± 1.82 ^a^	1.68 ± 0.97 ^a,b^	3.05 ± 1.16 ^a^	0.53 ± 0.27 ^a^
C57BL/6 + PM_2.5_ (30)	435 ± 45 ^a,b^	1.47 ± 1.09 ^a,b^	1.63 ± 0.68 ^a,b^	3.56 ± 1.11 ^a,b^	0.45 ± 0.12 ^a,b^
Before instillation					
ApoE^−/−^ + saline	533 ± 27	5.33 ± 0.38	1.14 ± 0.09	2.44 ± 0.18	0.52 ± 0.02
ApoE^−/−^ + PM_2.5_ (3)	549 ± 35	5.9 ± 0.62	1.03 ± 0.02	2.75 ± 0.35	0.45 ± 0.07
ApoE^−/−^ + PM_2.5_ (10)	536 ± 40	5.75 ± 0.34	0.91 ± 0.39	2.47 ± 0.75	0.42 ± 0.06
ApoE^−/−^ + PM_2.5_ (30)	524 ± 44	5.98 ± 0.71	1.37 ± 0.10	2.54 ± 0.1	0.55 ± 0.02
After instillation					
ApoE^−/−^ + saline	530 ± 19	5.25 ± 1.05	0.97 ± 0.21	2.48 ± 0.45	0.46 ± 0.13
ApoE^−/−^ + PM_2.5_ (3)	536 ± 37	2.9 ± 0.42 ^a,b^	0.86 ± 0.24 ^c^	2.95 ± 0.28	0.54 ± 0.16 ^c^
ApoE^−/−^ + PM_2.5_ (10)	484 ± 47	2.82 ± 0.96 ^a,b^	0.73 ± 0.23	3.25 ± 0.85	0.21 ± 0.03 ^a,c^
ApoE^−/−^ + PM_2.5_ (30)	451 ± 39 ^a,b^	2.57 ± 0.41 ^a,b^	0.21 ± 0.04 ^b,c^	3.94 ± 0.75 ^b^	0.05 ± 0.01 ^a,b,c^

Table 3 Changes in heart rate and HRV indices in mice after exposed to PM_2.5_. The doses were 0 (control), 3, 10, and 30 mg/kg b.w. Significant difference (^a^ *p* < 0.05) between PM_2.5_ group and control group, significant difference (^b^ *p* < 0.05 ) between before instillation and after instillation by self-control, and significant difference (^c^ *p* < 0.05) between C57BL/6 and dose- matched ApoE^−/−^ mice were observed.

**Table 4:** Echocardiography changes in experimental groups after PM_2.5_ exposure.

Groups	EF	FS	LVES*d*	LVED*d*	LVPW*d*	LVAW*d*
C57BL/6 + saline	57.21 ± 12.11	30.38 ± 8.16	3.14 ± 0.73	4.46 ± 0.63	0.69 ± 0.14	0.75 ± 0.12
C57BL/6 + PM_2.5_ (3)	54.04 ± 7.06	28.12 ± 4.73	3.4 ± 0.21	4.73 ± 0.30	0.62 ± 0.07	0.70 ± 0.09
C57 BL/6 + PM_2.5_ (10)	53.74 ± 13.40	28.19 ± 8.96	3.19 ± 0.56	4.43 ± 0.31	0.64 ± 0.12	0.69 ± 0.09
C57 BL/6 + PM_2.5_ (30)	50.21 ± 7.32	25.49 ± 4.69	3.28 ± 0.37	4.4 ± 0.24	0.57 ± 0.09	0.63 ± 0.08
ApoE^−/−^ + saline	67.69 ± 9.07	37.76 ± 7.34	2.57 ± 0.45	4.1 ± 0.28	0.74 ± 0.05	0.87 ± 0.18
ApoE^−/−^ + PM_2.5_ (3)	72.57 ± 6.93 ^a,b^	41.49 ± 6.37 ^a,b^	2.27 ± 0.43	3.84 ± 0.38	0.70 ± 0.07	0.78 ± 0.06
ApoE^−/−^ + PM_2.5_ (10)	74.76 ± 8.20 ^a,b^	43.16 ± 6.75 ^a,b^	2.52 ± 0.61	3.89 ± 0.66	0.77 ± 0.07	0.84 ± 0.08
ApoE^−/−^ + PM_2.5_ (30)	67.67 ± 12.25	37.97 ± 9.35	2.51 ± 0.75	3.98 ± 0.65	0.80 ± 0.18 ^a^	0.96 ± 0.30 ^a^

Table 4 Echocardiography changes in experimental groups after exposed to PM_2.5_. The doses were 0 (control), 3, 10, and 30 mg/kg b.w., respectively. Significant difference (^a^ *p* < 0.05) between PM_2.5_ group and control group, significant difference (^b^ *p* < 0.05) between C57BL/6 and dose-matched ApoE^−/−^ mice.

## Abbreviations

ApoE-/-	apolipoprotein E knock out
ECG	electrocardiogram
Echo	echocardiogram
HF	high frequency
HRV	heart rate variability
LBBB	left bundle-branch block
LF	low frequency
LV	left ventricle
LVEDd	left ventricular end diastolic dimension
LVESd	left ventricular end-systolic dimension
LVEF	left ventricular ejection fraction
LVFS	left ventricular fractional shortening
LVPWd	left ventricular end-diastolic posterior wall thickness
LVAWd	left ventricular end diastolic anterior wall thickness
MDA	malondialdehyde
NADPH	nicotinamide adenine dinucleotide 2′-phosphate
p22phox	nadph subunit 22-kilodalton
p47phox	nadph subunit 47-kilodalton
PM_2.5_	fine particulate matters
SOD	superoxide dismutase
